# DNA methylation alterations of *AXIN2* in serrated adenomas and colon carcinomas with microsatellite instability

**DOI:** 10.1186/1471-2407-14-466

**Published:** 2014-06-25

**Authors:** Yuta Muto, Takafumi Maeda, Koichi Suzuki, Takaharu Kato, Fumiaki Watanabe, Hidenori Kamiyama, Masaaki Saito, Kei Koizumi, Yuichiro Miyaki, Fumio Konishi, Sergio Alonso, Manuel Perucho, Toshiki Rikiyama

**Affiliations:** 1 Department of Surgery, Saitama Medical Center, Jichi Medical University, 1-847, Amanuma-cho, Omiya-ku, Saitama 330-8503, Japan; 2 First Department of Surgery, Hamamatsu University School of Medicine, 1-20-1, Handa-yama, Higashi-ku, Hamamatsu, Shizuoka 431-3192, Japan; 3 Nerima-Hikarigaoka Hospital, 2-11-1, Hikarigaoka, Nerima-ku, Tokyo 179-0072, Japan; 4 Sanford-Burnham Medical Research Institute (SBMRI), 10901 North Torrey Pines Road, La Jolla, California, USA; 5 Institute of Predictive and Personalized Medicine of Cancer (IMPPC), Carretera de Can Ruti S/N, 08916 Badalona, Barcelona, Spain; 6 Instituciò Catalana de Recerca i Estudis Avançats (ICREA), Passeig Lluis Companys 23, Barcelona, Spain

**Keywords:** Colon sessile serrated adenoma, Microsatellite instability, *BRAF* mutation, DNA methylation, *AXIN2*

## Abstract

**Background:**

Recent work led to recognize sessile serrated adenomas (SSA) as precursor to many of the sporadic colorectal cancers with microsatellite instability (MSI). However, comprehensive analyses of DNA methylation in SSA and MSI cancer have not been conducted.

**Methods:**

With an array-based methylation sensitive amplified fragment length polymorphism (MS-AFLP) method we analyzed 8 tubular (TA) and 19 serrated (SSA) adenomas, and 14 carcinomas with (MSI) and 12 without (MSS) microsatellite instability. MS-AFLP array can survey relative differences in methylation between normal and tumor tissues of 9,654 DNA fragments containing all *NotI* sequences in the human genome.

**Results:**

Unsupervised clustering analysis of the genome-wide hypermethylation alterations revealed no major differences between or within these groups of benign and malignant tumors regardless of their location in intergenic, intragenic, promoter, or 3′ end regions. Hypomethylation was less frequent in SSAs compared with MSI or MSS carcinomas. Analysis of variance of DNA methylation between these four subgroups identified 56 probes differentially altered. The hierarchical tree of this subset of probes revealed two distinct clusters: Group 1, mostly composed by TAs and MSS cancers with *KRAS* mutations; and Group 2 with *BRAF* mutations, which consisted of cancers with MSI and *MLH1* methylation (Group 2A), and SSAs without *MLH1* methylation (Group 2B). *AXIN2*, which cooperates with APC and β-catenin in Wnt signaling, had more methylation alterations in Group 2, and its expression levels negatively correlated with methylation determined by bisulfite sequencing. Within group 2B, low and high *AXIN2* expression levels correlated significantly with differences in size (P = 0.01) location (P = 0.05) and crypt architecture (P = 0.01).

**Conclusions:**

Somatic methylation alterations of *AXIN2*, associated with changes in its expression, stratify SSAs according to some clinico-pathological differences. We conclude that hypermethylation of *MLH1*, when occurs in an adenoma cell with *BRAF* oncogenic mutational activation, drives the pathway for MSI cancer by providing the cells with a mutator phenotype. *AXIN2* inactivation may contribute to this tumorigenic pathway either by mutator phenotype driven frameshift mutations or by epigenetic deregulation contemporary with the unfolding of the mutator phenotype.

## Background

Recent advances in colon cancer research have revealed a new pathological pathway distinct from the traditional pathway, the tubular adenoma-carcinoma sequence [1]. This alternative pathway has been recognized as the serrated pathway, in which sessile serrated adenoma (SSA) replaced the traditional tubular adenoma as the precursor lesion of a subset of colorectal cancer [2].

SSA was identified as a new entity by Torlakovic *et al.* in 1996 [3] and later classified in a new category, the serrated polyps [2]. The serrated polyps include hyperplastic polyps, traditional serrated adenomas and sessile serrated adenomas, the characteristics of which are serrated structure in the crypt epithelium [4-6]. Serrated polyp nomenclature is evolving and interpretation of the literature is complicated by differing interpretations of the morphological features of serrated polyps. Even among expert gastrointestinal pathologists there is significant inter-observer variability in classification [7,8].

Regardless of the difficulty in the definition, recent research efforts led to recognize that serrated polyps, especially SSA seemed to be precursor to many of the sporadic colorectal cancers with microsatellite instability (MSI) [9]. Mismatch repair deficiency leads to the accumulation of hundred of thousands of somatic mutations in microsatellite sequences [10]. This mutator phenotype defined a specific molecular pathway for colon cancer because the mutated cancer genes are in general different than those from cancers without MSI [10,11]. SSAs and MSI cancers were reported to exhibit similar features including predominant location in the proximal colon, high *BRAF* and low *KRAS* mutation and enhanced DNA hypermethylation [12-17].

Somatic hypermethylation of CpG islands in some genes includes the silencing of the *MLH1* mutator gene and thus underlies many of the MSI sporadic cancers. Some investigators conferred distinctive phenotypic and biological properties to the tumors displaying a so-called CpG island methylator phenotype (CIMP), which was viewed as preceding the development of a subset of MSI colon cancers [18,19]. However, no apparent bimodal distribution was seen for the somatic hypermethylation alterations in gastrointestinal cancers [20,21] thus challenging the CIMP hypothesis. Nearly 15 years later, the CIMP concept, despite the publication of many CIMP papers (reviewed in [22]) still awaits for a clear definition, including a stable set of CIMP markers, as well as for identification of the underlying methylator gene(s) [22,23].

Despite of the elusive CIMP entity, the importance of somatic hypermethylation as responsible for the silencing of several tumor suppressors and the *MLH1* mutator gene, and as a consequence the resulting MSI mutator phenotype, is highlighted by the evidence that SSA display DNA methylation alterations that are frequently observed in MSI cancer [9,24-26]. However, comprehensive analyses of methylation alterations in SSA and MSI cancer have not been conducted.

Methylation sensitive amplified fragment length polymorphism (MS-AFLP) is a fingerprinting technique developed by Yamamoto *et al*. as a tool to analyze DNA methylation in hundreds of *loci* simultaneously [27,28]. The approach utilized *NotI* restriction endonuclease for targeting methylation changes in any of the two CpG sites within its recognition sequence GCpGGCCpGC. Because nearly half of all *NotI* sites (44%) are located in or adjacent to CpG islands, while the rest are located outside, MS-AFLP enabled to detect both relative DNA hypermethylation and hypomethylation somatic alterations throughout the genome. Comparing the intensity of the fingerprint bands from normal and tumor tissue DNA provided an unbiased insight of the complex picture of those epigenetic alterations. Employing this technique for the study of colorectal cancer we demonstrated that the MSI phenotype was dominant over hypermethylation [21] and that some of the tumors without MSI could be rationalized by an age-associated accumulation of DNA hypomethylation [23].

More recently, we developed a novel MS-AFLP array-based platform containing probes consisting of 60-mer-oligonucleotides, which cover the sequences adjacent to all the 9645 *NotI* sites identified in the human genome [29]. In this study, we performed a comprehensive analysis of methylation alterations to characterize the epigenetic profiles of colon adenomas and carcinomas of different genotype and phenotype to identify genes shared by these different neoplasms.

## Methods

### Patients and tissues

Nineteen patients with sessile serrated adenoma (SSA), 8 with tubular adenoma (TA) and 26 with proximal colon cancer including 12 and 14 tumors with and without MSI, respectively were recruited in this study. These were from the series analyzed in our previous study, which had enough amount and high quality of DNA and RNA available for microarray analysis [17]. SSAs, TAs and colorectal cancer tissues were prospectively collected in Jichi Medical University Hospital and Jichi Medical University Saitama Medical Center. SSA and TA were obtained endoscopically and classified with two categories by the location, i.e., proximal and distal.

SSA was diagnosed by five architectural features; basal crypt serration, basal dilatation of the crypts, crypts that run horizontal to the basement membrane, crypt branching and surface villosity or papillarity as previously described [17,30-32]. When the endoscopically resected polyp exhibited two or more features was diagnosed as SSA. Lesions showing typical histological features of so-called “traditional serrated adenoma” [5] were excluded from the analysis.

Colon cancer tissues were obtained from patients who underwent surgical treatment. In all of the lesions, a part of the tissue was taken in fresh and was frozen immediately for genetic analysis and the rest of the tissue was used for histological analysis. Proximal lesions were defined as proximal to splenic flexure, whereas distal lesions were defined as distal to splenic flexure. All colorectal cancer tissues were collected from proximal colon. Written informed consent for participation in the study was obtained from all participants. This study was approved by Jichi Medical University Institutional Review Board.

### DNA and RNA extraction

DNA was extracted by DNeasy® blood and tissue kit (Qiagen, Hilden, germany). Total RNA was extracted from tissue culture cell lines by TRIzol® Plus RNA purification kit (Invitrogen, Carlsbad, CA, USA).

### *BRAF* and *KRAS* mutation analysis

*BRAF* (T1799A) and *KRAS* mutations were determined by direct sequencing after polymerase chain reaction (PCR) amplification of exon 15 of the *BRAF* gene and codon 12 and 13 of the *KRAS* gene. For detection of the *BRAF* mutation, genomic DNA obtained from fresh frozen samples was amplified using: forward, 5′-TCATAATGCTTGCTCTGATAGGA-3′ and reverse, 5′-GGCCAAAAATTTAATCAGTGGA-3′ primers. For the detection of the *KRAS* mutation, the following primers were used: forward, 5′-CTGAAAATGACTGAATATAAACTTGT-3′ and reverse, 5′-ATATGCATATTAAAACAAGATTTACC-3′ as described [17,33,34]. PCR products were purified on a YM-30 Microcon column (Millipore) and sequenced using the BigDye terminator v3.1 cycle sequencing kit on ABI Prism 3100 (both from Applied Biosystems, Tokyo).

### MSI analysis

Genomic DNA was extracted from fresh frozen samples using the EZ1 DNA tissue kit (Qiagen, Tokyo, Japan) and was amplified by PCR using the monomorphic markers BAT25 and BAT26 as previously described [27]. PCR products were analyzed by Gene Scan using ABI Prism 3100, and the sample was scored showing MSI if there were additional peaks in the PCR products, or otherwise scored as microsatellite stable (MSS).

### *MLH1* and CpG island methylation

Combined bisulfite restriction analysis was performed to assess gene methylation using primers that were designed to amplify the regions around the transcription start sites of the target genes [35]. Bisulfite modification was performed using the Epitect Bisulfite kit (Qiagen), as described previously [36]. Genomic DNA (1 μg) was used for conversion with the bisulfite reagent. The primer sequences, annealing temperatures and restriction enzymes utilized were identical to those previously described [37]. After digestion, products were electrophoresed on 2% agarose gels and stained with ethidium bromide. Methylation density was confirmed using the image analysis program Image J, and positive methylation was defined when the methylation- sensitive restriction enzyme digested ≥10% of the DNA [37].

### Preparation, labeling and hybridization of DNA samples for MS-AFLP arrays

The genome-wide methylation profile was determined by a high-throughput array-based analysis of methylation alterations. For this purpose, we introduced an array-based approach of the methylation sensitive amplified fragment length polymorphism (MS-AFLP) fingerprinting method, which can survey most of the 9654 DNA fragments containing all *NotI* sequences in the genome, as previously described [27,38]. Genomic DNA was isolated by QIAamp DNA Mini Kit (QIAGEN, Hilden, Germany). The initial steps of the MS-AFLP were performed as previously described [27,38]. Briefly, 1 μg of genomic DNA was digested overnight with 5 units of methylation-sensitive *NotI* (Promega, Madison, WI, USA) and 2 units of methylation-insensitive *Mse* I (NE Biolabs, Beverly, MA, USA) at 37°C. Two pairs of oligonucleotides were annealed overnight at 37°C to generate *NotI* (5′-CTCGTAGACTGCGTAGG-3′ and 5′-GGCCCCTACGCAGTCTAC-3′) and *Mse* I (5′-GACGATGAGTCCTGAG-3′ and 5′-TACTCAGGACTCAT-3′) specific adaptors.

The digested DNA was ligated in 1.25 μl each of 5 pmol/μl *NotI* and 50 pmol/μl *Mse* I adaptor using 1 unit of T4 DNA ligase (Promega) overnight at 16°C. The adaptor-ligated template DNA was amplified by PCR using *NotI* (5′-GACTGCGTAGGGGCCGCG-3′) and *Mse* I (5′-GATGAGTCCTGAGTAA-3′) primers. The PCR mixture consisted of 6 ng of *NotI* primer, 30 ng of *Mse* I primer, 0.25 mM dNTP, and 1.5 unit of AmpliTaq DNA polymerase (Applied Biosystems, Foster City, California, USA) in a final volume of 20 μl. The PCR started at 72°C for 30 s and 94°C for 30 s, followed by 35 cycles of 94°C for 30 s, 52°C for 30 s, and 72°C for 2 min. The final extension was performed for 10 min at 72°C. The reactions were then kept at 10°C until the amplified DNA fragments were isolated using a QIA PCR Clean-up kit (Qiagen). DNA was eluted into 50 μl of elution buffer.

Prior to hybridization on the MS-AFLP arrays, the DNA samples were differentially labeled as previously described [27,38]. Briefly, fluorescently labeled fragments were prepared using the Bioprime labeling system (Invitrogen). Each sample of PCR-amplified DNA (50 ng/2.5 μl) was mixed with 5 μl of water and 5 μl of Random Primer Mix solution. The mixtures were boiled at 100°C for 2 min, quickly placed on ice for 1 min, and briefly centrifuged for 10 s. Then 1 μl of either CY5 Mix solution (1.56 mM each of dGTP, dATP and dTTP, 0.22 mM dCTP, and 0.11 mM Fluorolink CY5-dCTP) or CY3 Mix solution (1.56 mM each of dGTP, dATP, and dTTP, 0.22 mM dCTP, and 0.11 mM Fluorolink CY3-dCTP) was added. Fluorolink CY5-dCTP and CY3-dCTP were purchased from Amersham-Pharmacia. Klenow fragment of *E. coli* DNA polymerase was then added to a final concentration of 0.8 U per μl. The mixtures were incubated at 37°C for 1 h before adding 2 μl of stop solution (0.5 M EDTA) to terminate the reaction.

The CY5 and CY3 fluorescently labeled DNA fragments were separated from the unincorporated dNTPs by filtration through Microcon YM-30 columns (Millipore, Bedford, MA, USA). Each sample was reconstituted with 1× TE (pH 8.0) to a final volume of 37 μl, and 2 μl of each sample was taken to determine the yield of labeled genomic DNA and the specific activity after labeling and clean-up. Exposure of samples to light was minimized during all experimental procedures.

The Cy3 and Cy5 labeled DNA samples were mixed in a siliconized tube with 70 μl of Agilent 2× Hi-RPM Buffer (Agilent, Santa Clara, CA, USA). The mix was heated at 95°C for 3 min and centrifuged at 6000 × g for 1 min to collect the sample at the bottom of the tube. One hundred and ten μl of hybridization sample mixture was applied slowly to the gasket slide into the Agilent SureHyb chamber base. Then, one microarray slide was placed onto the gasket slide, with the active side facing down. The SureHyb chamber was covered onto the slides, and the clamp assembly was slid onto both pieces. The assembled slide chamber was placed in a rotator rack inside a hybridization oven and rotated at 20 rpm and hybridized at 65°C for 40 hours. After hybridization, array slides were washed with Oligo aCGH Wash Buffer 1 at room temperature for 5 minutes and Oligo aCGH Wash Buffer 2 at 37°C for 1 min. To prevent Cy5 degradation by ozone, the slides were washed with acetonitrile for 30 seconds and then with Stabilization and Drying Solution for 30 seconds. The arrays were scanned using an Agilent G2565BA DNA Microarray Scanner.

### Quantitative reverse transcription-PCR

Tissue specimens were immediately added to RNA later (Ambion, Austin, TX, USA) and stored at -80°C until DNA or RNA extraction. Total RNA was immediately treated with DNase I (Invitrogen, Carisbad, CA, USA) and reverse-transcribed using a Superscript II reverse transcriptase kit (Invitrogen) to prepare first-strand cDNA. The primer sequences for *AXIN2* were 5′-CTGGCTCCAGAAGATCACAAAG-3′ (forward) and 5′-ATCTCCTCAAACACCGCTCCA-3′ (reverse). Thermal cycling conditions were 42°C for 60 min (cDNA synthesis), 95°C for 30 sec (hot start), and then 40 cycles of 95°C for 5 sec, 60°C for 30 sec, and 72°C for 60 sec. The expression level of *AXIN2* was determined using the fluorescence intensity measurements from the ABI 7900HT Real-Time PCR System Data Analysis Software. An *ACTB* fragment was amplified as an internal control.

### Bisulfite sequencing analysis

DNA sequencing was performed after bisulfite modification, as previously described [36]. The primers for the bisulfite sequencing were 5′-TTGTATATAGTTTAGYGGTTGGG-3′ (forward) and 5′-AAATCTAAACTCCCTACACACTT -3′ (reverse). PCR was performed for 45 cycles, consisting of denaturation at 95°C for 30 sec, annealing at 58°C for 30 sec, and extension at 72°C for 60 sec, followed by a final 7-min extension at 72°C for all primer sets. The sequences were subjected to a BLAST search to determine their location in the genome.

### Statistics

Fisher’s exact was used to examine associations between two categorical variables. Continuous variable comparisons between two groups were performed with the Student’s t-test for those variables following a normal distribution, or with the non-parametric Mann–Whitney-Wilcoxon test for those variables that do not follow a normal distribution. The level of statistical significance was set at *P* < 0.05, unless otherwise specified. To determine the significant genes from multiple samples, variance of analysis (ANOVA) with Bonferroni correction was carried out using MeV [39], by which hierarchical clustering sample and gene trees were also drawn, simultaneously. The threshold of significance was determined by Bonferroni correction set at *P* = 0.05. To account for the bias due to the partial gene representation in the MS-AFLP Array, all the gene enrichment analyses were performed using the list of the genes present in the array as a background, instead of the total number of genes in the human genome [38].

## Results

### Clinicopathological and molecular features of samples

The clinicopathological and molecular features of the four subgroups of tumors, tubular adenomas (TA) sessile serrated adenomas (SSA), and MSI and MSS carcinomas, are summarized in Table 1. Patient gender and stage were not significantly different between each group. Patients with MSI cancers were older than those with SSA (P = 0.01). MSI associated with poorly differentiated phenotype. *KRAS* mutation was more prevalent in MSSs and TAs as compared to MSIs and SSAs (P < 0.01), whereas *BRAF* mutation was preferentially observed in MSIs and SSAs as compared to MSSs and TAs (P < 0.01). *MLH1* methylation was detected only in MSI carcinomas.

**Table 1:** Clinicopathological and molecular data of colon adenomas and carcinomas

	**TA (n = 8)**	**SSA (n = 19)**	**MSS (n = 12)**	**MSI (n = 13)**	**P-value**^**3**^
**Age**	65.4 ± 4.4	60.6 ± 9.8	62.2 ± 8.2	70.8 ± 11.3	**0.019**^**(a)**^
**Gender (M/F)**	4/4	13/6	6/6	6/7	0.57
**Duke’s stage**	NA	NA	7/5	11/2	0.20
**(A or B/C)**					
**Grade**^**1**^	NA	NA	12/0	9/4	0.096
**(W-M/P)**					
***KRAS *** **Mutations**	4/8	0/19	4/12	0/13	**0.0011**
**(mut/total)**					
***BRAF *** **Mutations**	0/8	19/19	4/12	9/13	**2.2×10**^**-6**^
**(mut/total)**					
***hMLH1 *** **Methylation (+/total )**	0/8	0/19	0/12	5/13	**8.6×10**^**-4**^
**Hypermethylation**^**2**^	1.8 ± 1.4%	1.2 ± 0.6%	1.2 ± 0.6%	1.9 ± 1.2%	0.15^(a)^
**Hypomethylation**^**2**^	0.6 ± 0.4%	0.3 ± 0.2%	1.0 ± 0.5%	1.0 ± 0.6%	**5.8×10**^**-4**^^**(a)**^

^1^Tumor grade. Well (W), moderately (M) or poorly (P) differentiated.

^2^Hypermethylation and hypomethylation indicate the percentage of MS-AFLP array probes with values surpassing the hypermethylation and hypomethylation thresholds, respectively.

^3^For categorical data, p-values were calculated by χ^2^ test when comparing four groups, or by Fisher’s exact test when comparing two groups. For continuous data, we applied one-way ANOVA followed by Tukey’s HSD multi-hypothesis testing correction. The most Statistically significant p-value after correction corresponded always to the SSA vs MSI comparison^(a)^. In hypomethylation, a significant difference between SSA and MSS was also found (P = 0.0014). P-values below 0.05 are in bold type.

NA: Not applicable.

**Figure 1:**
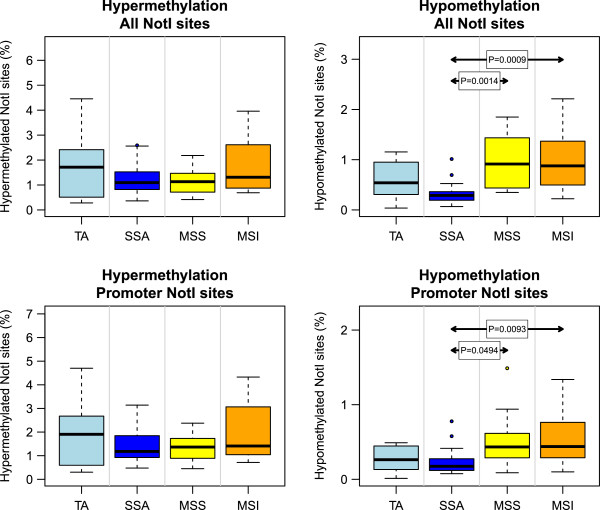
**Frequency of hypermethylation (left) and hypomethylation (right) estimated by MS-AFLP arrays.** TA, in light blue: tubular adenomas. SSA, in dark blue: sessile serrated adenomas. MSS, in yellow: microsatellite stable carcinomas. MSI, in orange: microsatellite instable carcinomas. Frequencies were calculated as the percentage of probes with log2 ratio value below -1.5 (for hypermethylation) or above 1.5 (for hypomethylation), after filtering the 30% lower-intensity probes from each array. Top graphs, results including all probes after filtering (13,515 probes per array). Bottom graphs, results considering only the probes within ±2.5 Kb of the 5′ end of genes (range: 7,924 to 8,035 probes per array). P-values were calculated by one-way ANOVA followed by Tukey’s HSD multi-hypothesis testing correction. Only p-values below 0.05 are shown.

Genome-wide surveillance of methylation alterations by MS-AFLP revealed no significant differences between the 4 groups (Table 1). Also, there was no significant difference in the overall frequency of hypermethylation alterations (including intragenic and intergenic regions) and in the promoter and 3′end regions (Figure 1). There was a borderline difference in methylation frequency at intragenic and 3′end regions (P = 0.035 and P = 0.041, respectively) between SSAs and MSI carcinomas due to the higher number of alterations of the later (Additional file 1: Figure S1). Regarding hypomethylation, SSAs displayed fewer alterations than MSS and MSI carcinomas overall and in the different gene regions although the differences were more pronounced compared with the MSI carcinomas (Figure 1 and Additional file 1: Figure S1).

Since the MS-AFLP array covers all *NotI* sites in the genome, these results extend our previous findings with the MS-AFLP DNA fingerprinting lower resolution approach showing that the original method reflected a panoramic view of the somatic methylation alterations undergone by colon cancers at *NotI* sites.

### Differentially methylated loci in TA, SSA, MSS and MSI

**Figure 2:**
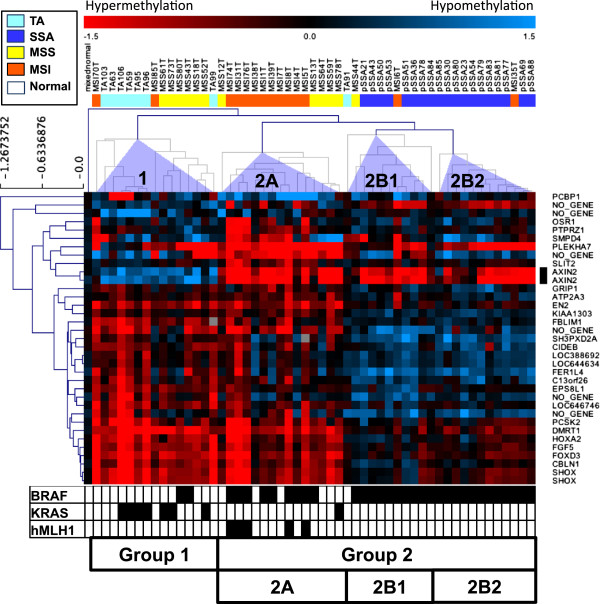
**Clustering of the samples according to their methylation profile.** Hypermethylation is indicated in red. Hypomethylation is indicated in blue. Samples are shown on top of the heatmap. In white, the normal tissue DNA mix used as reference. Colors for the four tumor groups are as in Figure 1. On the right side of the heatmap, the genes associated to MS-AFLP probes. *AXIN2* probes are indicated in black. Clustering was performed by complete linkage using Pearson’s correlation on a subset of MS-AFLP probes previously selected by ANOVA. Group 1 contains mostly tubular adenomas and MSS carcinomas. Group 2A contains the majority of MSI carcinomas, and 5 MSS carcinomas. All the sessile serrated adenomas are grouped in 2B1 and 2B2. Below the heatmap, in black, cases positive for mutation in *BRAF* or *KRAS*, or hypermethylation of *hMLH1*.

To identify the possible existence of distinct methylation profiles specific for each of the four different tumor subgroups, unsupervised hierarchical clustering was performed using 9,645 probe sets, but the results revealed no clear differences (data not shown). We, then, carried out an analysis of variance (ANOVA) to determine whether there were particular *loci* specifically associated with these tumor subgroups, especially with serrated adenomas. This analysis resulted in the identification of 56 distinctive probes, corresponding to 35 genes, 5 putative loci and 12 intergenic sequences (Figure 2 and Additional file 1: Table S1) that appeared altered differentially among these subgroups.The ANOVA-constructed hierarchical tree revealed two distinct subsets of samples (Groups 1 and 2, Figure 2). Seven MSSs (58.3%) and 2 MSIs (14.3%) cancers, as well as 7 TAs (87.5%) were assigned to Group 1, while 12 MSIs (85.7%), all 19 SSAs (100%), 5 MSSs (41.7%) and 1 TA (12.5%) to Group 2. Group 1 thus, consisted of many tumors participating in the tubular adenoma-carcinoma pathway (TAs and MSSs cancers), whereas Group 2 included many SSAs and MSI cancers (Figure 2).

### Distinct methylation profiles associates with SSAs including *AXIN2*

The clustering into two distinct groups by the ANOVA approach allowed performing t-test analysis of these two groups (Additional file 1: Figure S2). The constructed hierarchical tree revealed distinct epigenetic profiles, one of which was shared by many of the MSI cancers and SSAs, and another, which was shared by MSS carcinomas and TAs. T-test also identified 168 probes that distinguished these tumors of the serrated-MSI cancer pathway and the tubular adenoma-MSS carcinoma pathway (Additional file 1: Figure S2). *AXIN2* was one of these genes, which displayed a distinct level of methylation alterations between the two groups (black bar at right margins of Figure 2 and Additional file 1: Figure S2).

Group 2 was further classified into two subgroups, one of which included most MSI cancers (71.4%, Group-2A), and the other which contained all the SSAs (100%, Group-2B) (Figure 2). Group 1 displayed high frequency of *KRAS* mutation whereas Group 2 exhibited high frequency of *BRAF* mutation (Figure 2 bottom). While Group 2 harbored many of MSI cancers and all SSAs, as expected, methylation of *MLH1* was only seen in MSI cancers in Group 2A. Group 2B was also subdivided into two groups, 2B-1 and 2B-2, according to the hierarchical tree (Figure 2 bottom).

### Expression of *AXIN2* associates with methylation alterations

**Figure 3:**
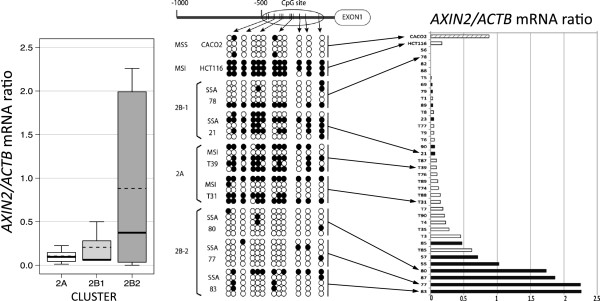
**Left: *AXIN2 *mRNA expression level of samples selected from cluster 2A, 2B1 and 2B2.** Expression levels were analyzed by Q-PCR using *ACTB* housekeeping gene for normalization. Thick horizontal lines within the boxes indicate the median value. Thin dashed horizontal lines indicate the mean value. Middle: bisulfite sequencing results of the 0.5Kb upstream region of the first exon of *AXIN2*. CpG sites are represented by a white or a black circles for unmethylated or methylated sites, respectively. Right: *AXIN2* mRNA expression level in colorectal cancer cell lines HCT116 (MSI, white bar) and Caco2 (MSS, stripped bar), and tumors, both sessile serrated adenomas (black bars) and MSI carcinomas (white bars), ordered from lower to higher (top to bottom). Arrows between middle and right panel connect those cases for which both the bisulfite sequencing and the mRNA expression results are shown in the figure.

ANOVA analysis identified *AXIN2*, which plays an important role in Wnt signaling pathway cooperating with APC and b-catenin, being more frequently altered in Group 2 that included the serrated adenomas. We measured the abundance of the corresponding *AXIN2* mRNA in the original SSAs and MSI cancers (Group 2) by quantitative reverse transcription–polymerase chain reaction (RT–PCR). The expression levels were variable, with some tumors showing little or no expression, while others exhibited a relatively high expression (Figure 3 right).

When the expression levels of *AXIN2* were compared between the different groups according to the hierarchical tree (Figure 2), the groups 2A and 2B-1 exhibited low expression levels of *AXIN2*, whereas group 2B-2 had significant higher levels of expression of *AXIN2* (Figure 3, left). To examine whether the decreased levels of *AXIN2* mRNA was linked to aberrant methylation, the degree of methylation alterations of 12 CpG sites within the *AXIN2* promoter region (Figure 3 middle) was assessed in 5 plasmid clones of each of several samples from groups 2A, 2B-1 and 2B-2. The *AXIN2* promoter appeared more methylated in samples from Group 2A than in Groups 2B-1 and 2B-2 (Figure 3 middle). Also, a tumor cell line with high methylation (HCT116) exhibited lower expression of *AXIN2* than another cell line (Caco2) with little methylation (Figure 3, top of middle and right panels).

### Expression of *AXIN2* associates with different clinicopathological features of SSAs

The clinicopathological features of SSAs were analyzed in regards to the observed differences in *AXIN2* methylation and expression of the two subgroups 2B-1 and 2B-2. The results are shown in Table 2. No differences were evident between these two groups in age, gender, presence of carcinoma in the adenoma, or mucinous phenotype. However, the adenomas with high methylation and low expression (Group 2B-1) were significantly smaller, were more distal, and exhibited less crypt branching than those adenomas with low levels of methylation and high levels of expression (Group 2B-2).

**Table 2:** Comparison of SSAs according to AXIN2 expression status

	**Group 2B-1 (n = 8)**	**Group 2B-2 (n = 11)**	**P-value**^**4**^
**Gender (Male/Female)**	4/4	9/2	0.32
**Patient Age (years)**	62.8 ± 9.9	59.1 ± 9.9	0.44
**(Range, Median)**	(46–74, 63)	(40–76, 59)	
**Tumor Size (mm)**	9.4 ± 1.9	15.3 ± 6.1	**0.01**
**(Range, Median)**	(6–12, 10)	(8–28, 15)	
**Location (C-A/T)**^**1**^	3/5	10/1	**0.041**
**Crypt Branching**^**2 **^**(+/total)**	0/8	8/11	**0.003**
**Hypermucinous**^**3 **^**(+/total)**	8/8	11/11	1.00
**Carcinoma in adenoma (+/total)**	0/8	1/11	1.00

^1^Anatomical location. Cecum (C). Ascending (A). Transversal (T).

^2^Crypt branching was defined by the appearance of splits or fissures in the base of the crypts.

^3^Hypermucinous appearance.

^4^P-values were calculated by Fisher’s exact test for categorical variables and unpaired t-test for continuous variables. P-values below 0.05 are in bold type.

## Discussion

In this study, genome-wide surveillance of hypermethylation alterations in *Not*I sites by MSFLP-array revealed that somatic hypomethylation was lower in SSAs compared with MSI or MSS carcinomas. These benign tumors also occurred in younger individuals compared with MSI carcinomas. This is consistent with the proposed hypothesis of demethylation as a gradual accumulation of methylation replication errors during aging [23] assuming SSAs being the precursors of the MSI carcinomas. In contrast, there were no major differences in global hypermethylation between these groups of benign and malignant tumors regardless of their location in intergenic, intragenic, promoter, or 3′end regions. Unsupervised clustering analysis revealed no clear differences in the patterns of hypermethylatuon between or within the four different tumor groups. Only after applying an ANOVA approach was possible to discern that MSS cancers and TAs shared similar epigenetic features, so did MSI and SSA, as reported previously [12-17]. The study also disclosed distinct profiles of genes relevant for colorectal cancer such as homeobox genes, transcription factors, growth factors and genes in the Wnt signaling pathway, including *AXIN2.*

Several papers estimated the frequency of Wnt signaling activation in SSAs but they are controversial [40-44]. Possible explanations to account for the discrepancies may include that some SSAs were misdiagnosed and wrongly categorized due to the complication in the definition of serrated polyps [45]. Therefore, a standardized diagnosis of SSA formulated recently [30,32] was applied in this study.

Recent genome-scale exome sequencing analysis of 276 colorectal tumors, DNA copy number, promoter methylation and messenger RNA and microRNA expression conducted by the Cancer Genome Atlas project, [46] indicated that 92% of MSI cancer and 97% of MSS cancers exhibited at least one alteration of genes involved in the Wnt pathway including *LRP5*, *FZD10*, *FAM123B*, *AXIN2*, *APC*, *CTNNB1* (β-catenin), *TCF7L2*, *FBXW7* and *SOX7*. Thus, Wnt signaling pathway seems to play a critical role in colorectal carcinogenesis in general, although the spectrum of alterations may vary depending on the distinct oncogenic pathways.

AXIN was identified as a component of the complex in Wnt signaling pathway to regulate the levels of β-catenin along with the wild type of adenomatous polyposis coli (*APC*) gene [47]. AXIN1 plays as a scaffold protein on which the complex for phosphorylation of β-catenin by glycogen synthase kinase-3β (GSK-3β) is assembled [48]. *AXIN2* / Conductin was identified as an AXIN homolog, which also played a scaffold protein, and was found mutated in a subset of colorectal cancers [47,49]. *AXIN1* appears to be a constitutive component of β-catenin degradation complex for maintenance of basal life activity while *AXIN2* is considered to be an inducible component that is upregulated in response to increases in β-catenin levels and thus serves to limit the duration and intensity of the Wnt signal [50,51]. *AXIN2* has been only found expressed in colon tissues (Additional file 1: Figure S3).

Epigenetic silencing of *AXIN2* in MSI colon cancer was reported in 2006 [52]. However, aberrant methylation of *AXIN2* in SSA has not been previously reported. In addition, we identified an apparent increase in methylation alterations of *AXIN2* from SSAs to MSI carcinomas, suggesting that its expression deregulation by methylation associates with the serrated adenoma-MSI cancer pathway.

The hierarchical tree identified three clusters according to methylation profiles, MSI, SSAs epigenetically close to MSI and SSAs far from MSI. The expression levels of *AXIN2* in these three groups associated with the levels of methylation of *AXIN2* in each group, respectively (Figure 3). Our results suggest that expanding of methylation in the promoter region of *AXIN2* in SSAs lead to the suppression of the *AXIN2* gene expression gradually, which contributes to a stepwise acquisition of the epigenetic features seen in MSI colon cancer. Koinuma *et al.*[52] reported that overexpression of *AXIN2*, either by treatment with 5′-azacytidine or by transfection with *AXIN2* cDNA, resulted in rapid cell death in a MSI CRC cell line, which supports the functional significance of *AXIN2* changes in methylation and expression in our study. Dong *et al.*[24] reported progressive methylation of several genes during the serrated pathway. In contrast with the epigenetic silencing of *AXIN2* in MSI colon cancer, up-regulation of *AXIN2* mRNA was reported in MSS cancers. Indeed, in our study, *AXIN2* was frequently hypomethylated in MSS cancers, suggesting that the epigenetic change of *AXIN2* specifically associates with the MSI pathway for colon cancer. The fact that down-regulation is not always accompanied by methylation (Figure 3) shows that additional mechanisms may be at play to inactivate the suppressor function of the AXIN2 protein. For instance, frameshift mutations of *AXIN2* in MSI colon cancers may be one such additional mechanism [49,53,54].

The epigenetic influence on MSI manifestation is shown by the hypermethylation and silencing of *MLH1*[55]. High level of hypermethylation has been also associated with MSI cancers [19,56], and also in SSAs [9,13,15,16,24-26,57,58]. However, *MLH1* methylation was not detectable in SSAs in contrast with the common presence observed in MSI cancers. This shows that the epigenetic silencing of *MLH1* is not involved in SSA development where it must occur sometime during the adenoma expansion. But silencing of *MLH1* then appears to drive the adenoma cells towards the carcinoma state by the generation of many subsequent mutations. The difference in age between the patients with SSAs and MSI carcinomas also supports this suggestion, implying a necessary additional step after SSA development for the accumulation of oncogenic mutations responsible for the carcinoma transition.

*AXIN2* aberrant methylation appears to occur during adenoma growth like *MLH1* methylation. The assumption here is that no methylation of *MLH1* is found at the SSA stage because once it occurs it may lead to the carcinoma transition in the absence of further clonal expansion, since mutator genes do not alter the growth properties of the cells. The association observed between aberrant methylation and down-regulation with small size SSAs without crypt branching could be interpreted assuming that the occurrence of *MLH1* methylation may speed the transition to carcinoma in the absence of a need for further expansion of the adenoma.

## Conclusions

In conclusion, this study revealed that methylation aberrations likely play a role in the serrated adenoma-MSI carcinoma sequence in colon cancer. Although the samples in this study are too limited to draw definitive conclusions in some genetic or epigenetic comparisons, other differences were sufficiently large to reach statistical significance. *MLH1* silencing seem to occur in an already developed serrated adenoma by the previous occurrence of somatic mutation in the *BRAF* oncogene. Once the serrated adenoma has evolved, additional somatic alterations altering Wnt signaling, such as *AXIN2* methylation or frameshift mutation, may contribute to the adenoma’s further growth. Other genes besides *AXIN2*, were identified that exhibit methylation profiles shared between SSA and MSI CRC and would be interesting to further investigate how these genes work and interact with each other during the progression of colon cancer of the serrated adenoma-MSI carcinoma sequence. Nevertheless, when, contemporary with these somatic alterations, aberrant methylation of the *MLH1* gene occurs, this appear to be the determinant event in those cases that eventually progress to the carcinoma stage by providing the cells with a strong mutator phenotype.

## Competing interests

The authors declare that they have no competing interests.

## Authors’ contributions

Experimental design: Y Muto, TM, KS, FK, MP, TR. Pathology analyses: TM. Molecular analyses (i.e., MSI and methylation analyses, bisulfite sequencing, etc.) Y Muto, TM, KS, MS, TK, FW, HK. MS-AFLP Array design: KS, SA. MS-AFLP implementation, data acquisition and analysis: TM, KS, HK, MS, KK, Y Miyaki, SA, MP. Statistical analysis: Y Muto, KS, SA. Experimental work supervision and coordination: KS, FK, MP, TR. Interpretation of the data: Y Muto, TM, KS, TK, SA, MP, TR. Manuscript writing: Y Muto, TM, KS, TK, SA, MP. All authors read and approved the final manuscript.

## Pre-publication history

The pre-publication history for this paper can be accessed here:

http://www.biomedcentral.com/1471-2407/14/466/prepub

## Supplementary Material

Additional file 1: Figure S1 Frequency of hypermethylation and hypomethylation of the different tumor groups, estimated by MS-AFLP arrays. **Figure S2.** Clustering of the samples according to their methylation profile. **Figure S3.** Gene expression pattern of the *AXIN2* gene in human normal and cancerous tissues. **Table S1.***Loci* with differential methylation alterations in the tumor groups. Click here for file
