# Supplementary material for: DNA methylation alterations of AXIN2 in serrated adenomas and colon carcinomas with microsatellite instability
Source: BMC Cancer. 2014 Jun 25;14:466. doi: 10.1186/1471-2407-14-466 (PMC4099028; doi:10.1186/1471-2407-14-466)
Supplement: Additional file 1: Figure S1 — Frequency of hypermethylation and hypomethylation of the different tumor groups, estimated by MS-AFLP arrays. Figure S2. Clustering of the samples according to their methylation profile. Figure S3. Gene expression pattern of the AXIN2 gene in human normal and cancerous tissues. Table S1.Loci with differential methylation alterations in the tumor groups. [file 1471-2407-14-466-S1.pdf]

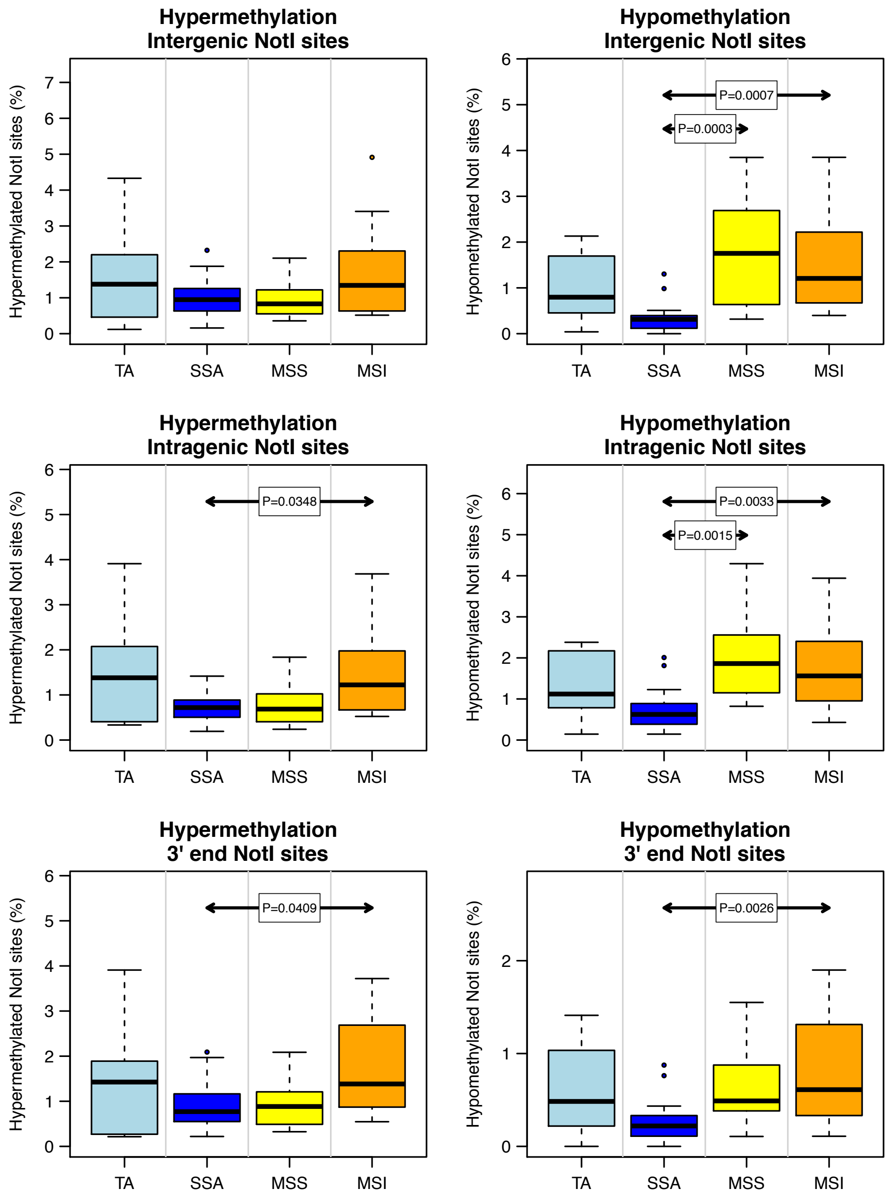

Supplementary Figure 1. Frequency of hypermethylation (left) and hypomethylation (right) of the different tumor groups, estimated by MS-AFLP arrays. TA, in light blue: tubular adenomas. SSA, in dark blue: sessile serrated adenomas. MSS, in yellow: microsatellite stable carcinomas. MSI, in orange: microsatellite instable carcinomas. Frequencies were calculated as the percentage of probes with log2 ratio value below -1.5 (for hypermethylation) or above 1.5 (for hypomethylation), after filtering the 30% lower-intensity probes from each array. Top graphs, results of probes in intergenic regions (range: 2,495-2,571 probes per array, depending on the sample). Middle graphs, probes inside gene body, defined as the region from 2.5Kb downstream the 5' end and the 3' end (range: 2,069-2,127 probes per array). Bottom graphs, probes within the 2.5Kb downstream the 3' of genes (range: 893-935 probes per array). P-values were calculated by one-way ANOVA followed by Tukey's HSD multi-hypothesis testing correction. Only p-values below 0.05 are shown.

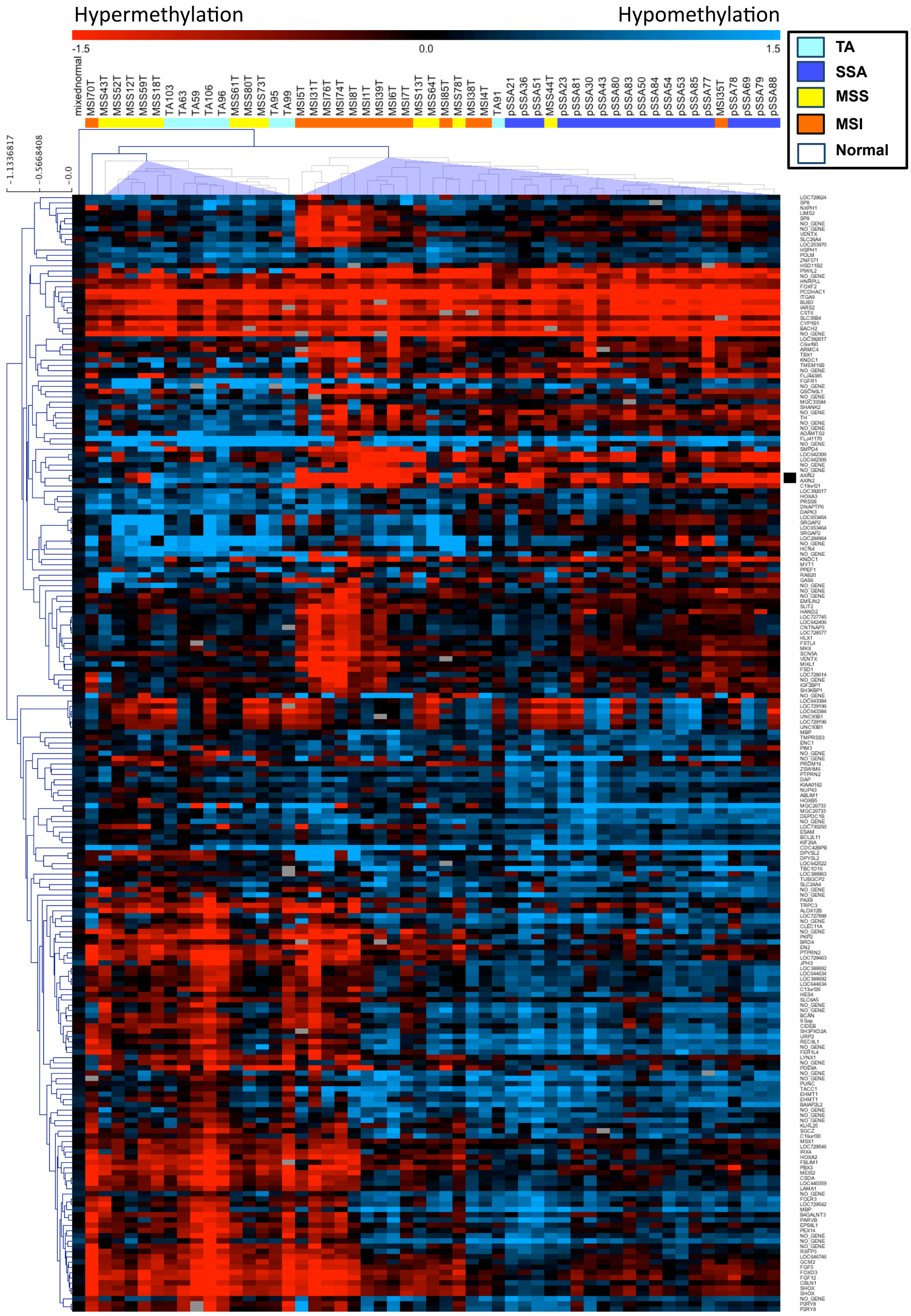

Supplementary Figure 2. Clustering of the samples according to their methylation profile. Hypermethylation is indicated in red. Hypomethylation is indicated in blue. Samples are shown on top of the heatmap. In white, the normal tissue DNA mix used as reference. TA, in light blue: tubular adenomas. SSA, in dark blue: sessile serrated adenomas. MSS, in yellow: microsatellite stable carcinomas. MSI, in orange: microsatellite instable carcinomas. On the right side of the heatmap, the genes associated to MS-AFLP probes. *AXIN2* probes are indicated in black. Clustering was performed by complete linkage using Pearson's correlation on a subset of MS-AFLP probes previously selected by t-test.

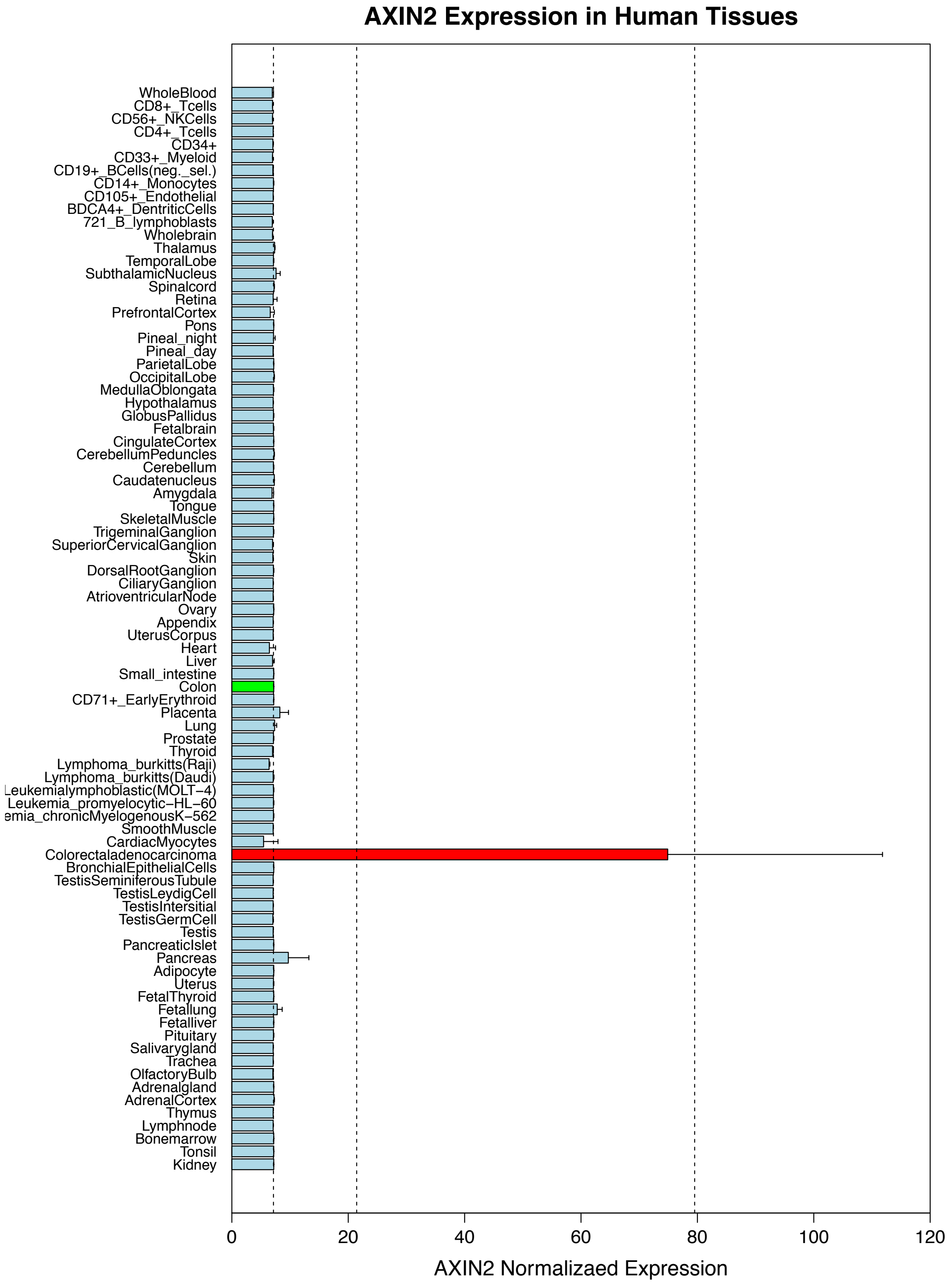

Supplementary Figure 3. Gene expression pattern of the *AXIN2* gene in human normal and cancerous tissues. *AXIN2* is dramatically overexpressed in colorectal adenocarcinomas (red bar) compared to normal colon tissue (green bar). Vertical dashed lines represente the 1x, 3x and 10x mean expression values. Diagram created for the gnf1h00059_at probe of the dataset GeneAtlas U133A, gcrma, obtained from BioGPS (http://biogps.org/dataset/1/). [1]

Supplementary Table 1. *Loci* with differential methylation alterations in the tumor groups.

Gene	Description	Function	Chrom.
**ATP2A3**	TPase, Na+/K+ transporting, alpha 3 polypeptide	ATPase, Na+/K+ transporting	17
AXIN2	*AXIN2*	inhibitor of the **Wnt** signalling pathway	17
**C13orf26**	Chromosome 13 open reading frame 26	unknown	13
**CBLN1**	Cerebellin 1 precursor	a cerebellum-specific precursor protein	16
**CSDA**	DNA-binding protein A	Shoch domain protein	9
**DMRT1**	Doublesex and mab-3 related transcription factor 1	transcription factor	9
**DOCK8**	Dedicator of cytokinesis 8	interact with guanine exchange factor **(GEF)**	12
**EN2**	Engrailed homeobox 2	**Homeobox**	7
**EPS8L1**	EPS8-like 1, transcript variant 1,	related to **EGFR** pathway substrate 8	19
**FBLIM1**	Filamin binding LIM protein 1, transcript variant 1	cell adhesion structures to the actin cytoskeleton	1
**FER1L4**	Fer-1-like 4 (C. elegans), non-coding RNA	non-coding RNA	20
**FGF5**	Fibroblast growth factor 5, transcript variant 1	member of the fibroblast growth factor **(FGF)** family	4
**FOXD3**	Forkhead box D3	forkhead family of transcription factors	1
**FSD1**	Fibronectin type III and SPRY domain containing	associates with a subset of **microtubules**	19
**FSTL1**	Follistatin-related protein 1	autoantigen associated with rheumatoid arthritis	3
**GRIP1**	Glutamate receptor interacting protein 1	glutamate receptor interacting protein family	12
**HOXA2**	Homeobox A2	**Homeobox**	7
**KIAA1303**	KIAA1199 (KIAA1303),	Regulatory-associated protein of **mTOR**	17
**OSR1**	Odd-skipped related 1	oxidative-stress responsive	2
**PCBP1**	Poly(rC) binding protein 1	retrotransposition of a fully processed PCBP-2	2
**PCSK2**	Proprotein convertase subtilisin/kexin type 2	subtilisin-like proprotein convertase family	20
**PDE9A**	High affinity cGMP-specific phosphodiesterase 9A	catalyzes the hydrolysis of cAMP and cGMP	21
**PKP2**	Plakophilin-2	may regulate **beta-catenin** signaling	12
**PLEKHA7**	Pleckstrin homology domain containing, family A member 7	pleckstrin homology domain containing	11
**POLM**	DNA polymerase mu	Involved in NHEJ pathway of DNA repair	7
**PRDM16**	PR domain containing 16	a zinc finger transcription factor	1
**PTPRZ1**	Protein tyrosine phosphatase, receptor-type, Z polypeptide 1	the receptor protein tyrosine phosphatase family	7
**REC8L1**	Human homolog of rad21	structural maintenance of chromosome)	14
**SEPT9**	Septin-9 **(methylated in colorectal cancer)**	essential for tumor cell migration and invasion	17
**SH3PXD2A**	SH3 and PX domains 2A	SH3 and PX domains 2A	10
**SHOX**	The paired **homeobox** family	**Homeobox**	X, Y
**SLIT2**	Slit homolog 2	interact with Glypican 1	4
**TACC1**	Transforming acidic coiled-coil-containing protein 1	breast cancer candidate gene	8
**URP2**	Fermitin family homolog 3 (FERMT3)	regulation of hemostasis and thrombosis	11
**VENTX**	Vent family of homeodomain proteins	**Homeobox**	10

The table lists in alphabetical order the *loci* found differentially methylated among the four different groups of tumors after ANOVA analysis. Of note the relative high frequency of cell growth signaling and transcription factors, including homeobox related loci, in consonance with our original prediction of high frequency of methylation alterations in homeobox genes [2].

Supplementary References

1. Wu C, Orozco C, Boyer J, Leglise M, Goodale J, Batalov S, Hodge CL, Haase J, Janes J, Huss JW, 3rd, Su AI: **BioGPS: an extensible and customizable portal for querying and organizing gene annotation resources.** *Genome Biol* 2009, **10:**R130.

2. Perucho M, Tokino T, Nakamura Y: **Cancer genomics and molecular diagnosis--The Nineteenth International Symposium of Sapporo Cancer Seminar.** *Jpn J Cancer Res* 1999, **90:**1273-1276.
